# Portable, multi-modal Raman and fluorescence spectroscopic platform for point-of-care applications

**DOI:** 10.1117/1.JBO.27.9.095006

**Published:** 2022-09-26

**Authors:** Cyril Soliman, Dandan Tu, Samuel Mabbott, Gerard Coté, Kristen Maitland

**Affiliations:** a Texas A&M University, Department of Biomedical Engineering, College Station, Texas, United States; b Texas A&M Engineering Experiment Station, Center for Remote Health Technologies and Systems, College Station, Texas, United States

**Keywords:** multi-modal, Raman spectroscopy, surface-enhanced Raman spectroscopy, fluorescence, point-of-care, spectroscopic platform

## Abstract

**Significance:**

Point-of-care (POC) platforms utilizing optical biosensing strategies can achieve on-site detection of biomarkers to improve the quality of care for patients in low-resource settings.

**Aim:**

We aimed to develop a portable, multi-modal spectroscopic platform capable of performing Raman and fluorescence measurements from a single sample site.

**Approach:**

We designed the spectroscopic platform in OpticStudio using commercial optical components and built the system on a portable optical breadboard. Two excitation and collection arms were utilized to detect the two optical signals. The multi-modal functionality was validated using ratiometric Raman/fluorescence samples, and the potential utility was demonstrated using a model bioassay for cardiac troponin I.

**Results:**

The designed spectroscopic platform achieved a spectral resolution of 0.67±0.2  nm across the Raman detection range (660 to 770 nm). The ratiometric Raman/fluorescence samples demonstrated no crosstalk between the two detector arms across a gradient of high molar concentrations. Testing of the model bioassay response showed that the integrated approach improved the linearity of the calibration curve from ($R^{2}=0.977$) for the Raman only and ($R^{2}=0.972$) for the fluorescence only to ($R^{2}=0.988$) for the multi-modal approach.

**Conclusion:**

These findings demonstrate the potential impact of a multi-modal POC spectroscopic platform to improve the sensitivity and robustness necessary for biomarker detection.

## 1 Introduction

Optical detection is a commonly used technique for probing tissue and blood samples to diagnose various health complications and diseases. Typically, benchtop systems within centralized laboratories process biological samples sent by local hospitals and clinics to aid in diagnostic screenings.^1^ In recent years, there has been an increase in miniaturized, point-of-care (POC) optical instruments to enable diagnostic capabilities within rural and underserved communities.^2^ A few examples of these POC technologies are Raman spectrometers, portable surface plasmon resonance platforms, and mini-potentiostats.^3^^–^^8^ The goal for developing improved POC devices in communities that lack access to centralized healthcare facilities is to provide faster screening and disease diagnosis through on-site sample analysis, which will improve the overall health outcomes for patients.

Fluorescence and Raman spectroscopy are two modalities that have been well integrated into POC devices. Fluorescence emission relies on the absorption of incident photons and reemission at longer wavelengths as molecules relax from an excited state back down to the ground state.^9^ Fluorescence is characterized by its high signal efficiency and broadband emission peaks. However, these two characteristics also limit its applications due to the overlap of the broad spectral peaks in the presence of multiple fluorophores and due to photobleaching that can occur following multiple or long exposures resulting in decreased signal efficiency.^9^ Raman spectroscopy is a vibrational spectroscopic technique that measures inelastic light scattering after excitation by incident photons. Based on the molecule’s chemical structure, different bonds within the molecule will vibrate, resulting in a measurable energy shift of the incident photons. Unlike the broad fluorescence emission peaks, Raman emission peaks are narrow and correspond to the specific vibrations of chemical bonds. Raman spectroscopy enables nondestructive fingerprint recognition of molecular samples with high detection accuracy and sensitivity. The main drawbacks of conventional Raman spectroscopy are the low signal magnitude (only about 1 in $10^{6}$ to $10^{8}\text{ photons}$ being inelastically scattered by a molecule) and lack of specificity due to the overlap of spectral information in complex media.^10^ Alternatively, surface-enhanced Raman spectroscopy (SERS) is an analytical technique that can enhance Raman scattering efficiency by exploiting localized surface plasmon resonance associated with metallic surfaces.^10^^–^^13^ The magnitude of SERS enhancement is due to various factors, including the metallic structure, material, and distance from the surface of the structure, resulting in reported enhancement factors ranging from $10^{6}$ to $10^{14}$.^10^^–^^13^ Metallic nanoparticles are a commonly utilized SERS substrate due to their manufacturing reproducibility, stability, and chemical functionalization properties.^10^^,^^12^ In addition, when select Raman reporter molecules (RRMs) are used, greater specificity can be obtained with SERS compared to regular Raman spectroscopy. However, signal repeatability is often a drawback with SERS methodologies.^14^^–^^16^

Both fluorescence and Raman optical modalities provide spectral information about a given sample; therefore, spectrometers are commonly used to measure emitted photons. Commercial spectrometer design has more recently focused on Raman applications. Specifically, Thermo Fisher Scientific, Ocean Insight, Wasatch Photonics, and B&W Tek have developed Raman spectrometers for medical applications, quality control, and monitoring. Other groups have focused on the development of portable Raman spectrometers, which can be more easily applied to in-field applications; however, addressing issues such as the reduction in large form factors, the requirement for a diverse array of accessories, complex interfaces requiring in-depth training, and cost remains a challenge.^17^^–^^31^ The main issue with developing a low-cost POC spectrometer is balancing device performance with the overall cost, size, and ease of functionality. Some low-cost spectrometers require mobile phones^17^^–^^21^ or a pump and probe geometry where the excitation and collection pathways are in separate modules.^22^^–^^25^ Both modalities can be employed together for medical imaging. In most cases, fluorescence is used for spatial localization within a region of interest, and Raman is used for acquiring specific biological identification.^32^^–^^36^^,^^37^ For example, Lee et al.^33^ used fluorescence for a fast indicator and used Raman to image the local distribution of CD 24 and CD 44 in breast cancer cells. Jeong et al.^35^ used fluorescence for the rapid localization of a pathologic lesion and used Raman for the identification of epidermal growth factor receptors. Within the context of diagnostic applications, both modalities report in a sample’s chemical content, leading to redundancy and enabling the potential for a built-in secondary validation metric. Zhang et al.^32^ used the dual modalities for detecting microRNA with improved sensitivity and accuracy. Some other applications of dual Raman and fluorescence include DNA and RNA detection, environmental toxin detection, and pH sensing. The development of dual Raman and fluorescence active sensing probes are well documented in the literature.^38^^–^^46^ However, to our knowledge, no commercially available small form factor multimodal spectrometer combines the benefits of fluorescence and Raman for POC monitoring. The spectroscopic platform proposed here integrates the excitation and collection optics into a single portable package with the flexibility to collect signals from a solid surface or liquid interface.^47^ In addition, the multi-modal functionality enables the collection of fluorescence and Raman scattered light from a single sample site. By leveraging the strengths of both optical modalities, we demonstrate that increased sensitivity and specificity can be achieved for diagnostic applications.

## 2 Materials and Methods

### 2.1 Design Considerations

Benchtop Raman spectrometers provide highly customizable platforms with multiple laser modules or gratings to meet application-driven device parameters. Benchtop spectrometers can achieve spectral resolution down to $1\;{\mathrm{cm}}^{-1}$ or 0.04 nm and are typically equipped with sample mapping capabilities. Comparably, with a restricted platform size, compact Raman spectrometers are designed to cover a narrow spectral bandwidth (400 to $2400\;{\mathrm{cm}}^{-1}$ or 100-nm bandwidth) with moderate spectral resolution (10 to $50\;{\mathrm{cm}}^{-1}$ or 0.4 to 2 nm). In comparison, spectrometers used for fluorescence measurements typically cover a larger spectral bandwidth (400 to 1000 nm) with relaxed spectral resolution requirements (10 nm). Conventionally, Raman spectra are plotted with wavenumbers (${\mathrm{cm}}^{-1}$) on the x-axis versus fluorescence plots that use wavelength (nm). *A priori* knowledge about the Raman spectra of the target analyte can drive the design parameters to ensure sufficient spectral resolution.^48^ A spectral resolution of 0.5 nm ($12\;{\mathrm{cm}}^{-1}$) over a spectral range of 100 nm (400 to $2560\;{\mathrm{cm}}^{-1}$) was selected as our design goal to resolve multiple peaks present in the Raman spectra of the cyanine 3 (Cy3) dye, which will act as our fluorophore and RRM. The proposed platform will have separate collection arms for the two optical modalities. The Raman collection arm will consist of a spectrometer, whereas an intensity-based measurement will be employed to capture the fluorescence signal to account for the differences in dynamic range requirements.

### 2.2 Spectrometer Design

The crossed Czerny–Turner optical configuration is commonly used for compact spectrometer design by leveraging two concave mirrors and a diffraction grating to fold the optical path. The crossed Czerny–Turner optical bench is typically used for applications that do not require high spectral resolution because the off-axis geometry results in relatively large aberrations. Lenses can also be used in place of the concave mirrors to reduce off-axis aberrations. The focal length of the collimating and focusing optics affects the overall spectral range covered by the spectrometer and the spectral resolution. Equations (1) and (2) estimate the relationship between the focal lengths of these optics and various optical parameters

$$L_{F}=\frac{L_{D}\;\cos(\beta )}{G(\lambda_{2}-\lambda_{1})},$$ (1)

$$L_{C}=L_{F}\frac{\cos(\alpha )}{M\;\cos(\beta )}.$$ (2)

Equation (1) relates the focal length of the focusing optics ($L_{F}$) to the angle of diffraction (β), the length of the detector ($L_{D}$), the grating groove density (G), and the wavelength range of interest ($\lambda_{2}-\lambda_{1}$). Equation (2) relates the focal length of the collimating optics ($L_{C}$) to the angle of incidence (α), the magnification (M), and the angle of diffraction. Based on the focal lengths of the optics selected to fit the design parameters, the slit width necessary to achieve the desired spectral resolution (Δλ) can be calculated using Eq. (3)

$$w_{\text{slit}}=\frac{G\Delta \lambda L_{C}}{\cos(\alpha )}.$$ (3)

**Fig. 1:**
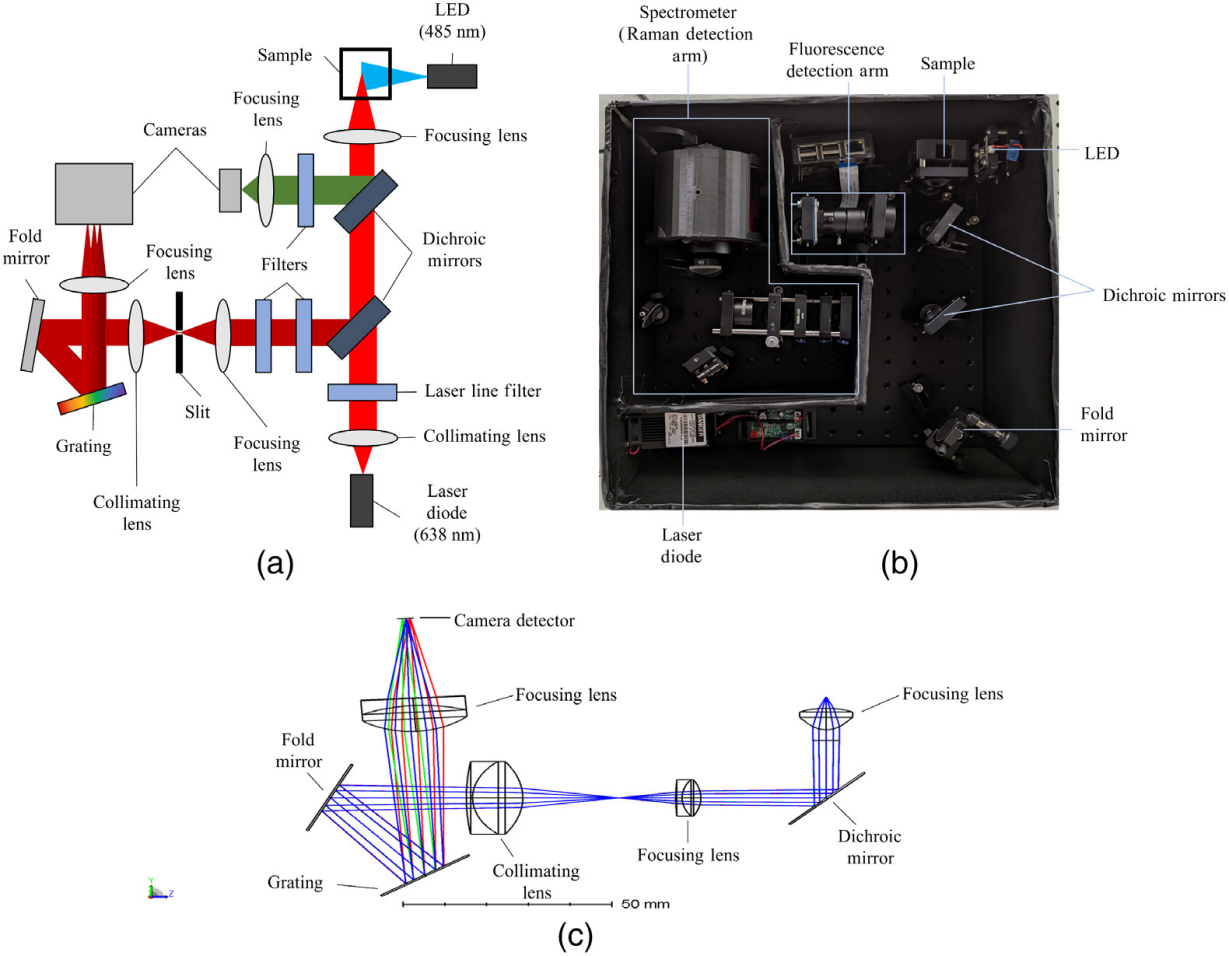
(a) Schematic of multi-modal optical system layout and (b) assembled optical system on a portable optical breadboard (12×12  in.). (c) Ray trace of the spectrometer and front optics portion of the optical system.

Equations (1)–(3) were used to select commercially available components capable of meeting the design requirements. The optical design of the crossed Czerny–Turner spectrometer layout was modeled using OpticStudio. Lenses were used instead of mirrors to minimize off-axis aberrations and improve ease of alignment. The collected light is directly coupled into the slit of the spectrometer without the need for optical fibers. This improves the percentage of Raman scattered light coupled into the spectrometer and minimizes the need for a costly detector. The optical system schematic, assembled optical system, and OpticStudio ray trace diagram for the crossed Czerny–Turner portion of the system are shown in Fig. 1.

### 2.3 Optical System Layout

The platform consists of separate excitation sources for each optical modality followed by separation of the fluorescence signal onto a complementary metal-oxide-semiconductor (CMOS) detector and Raman signal through the designed spectrometer containing off-the-shelf optical components and a CMOS detector. The Raman arm of the system is composed of a laser diode (L638P150, Thorlabs) centered at 638 nm (2.5-nm bandwidth) and collimated using an aspheric condenser lens (ACL12708U-A, Thorlabs) before passing through a laser line filter (FL05635-10, Thorlabs). The filtered laser beam passes through a short pass dichroic mirror (DMSP650, Thorlabs) before being focused onto the sample by an aspheric condenser lens (8-mm focal length, ACL108U-A, Thorlabs). The Raman emission is collected by the same lens, reflects off the dichroic mirror, and passes through two 650-nm long-pass filters (FELH0650, Thorlabs) to filter out any residual laser diode light leakage and Rayleigh scattered light. The two 650-nm long-pass filters were selected over a single high-efficiency notch filter to achieve comparable filtering efficiency at a lower cost. The beam is focused onto an adjustable slit (VA100/M, Thorlabs), set to 50  μm, using an achromatic doublet lens (AC127-019-B-ML, Thorlabs). The divergent beam is collimated by an achromatic doublet lens (AC127-030-B-ML, Thorlabs) into a fold mirror angled to redirect the collimated beam onto the 1800  grooves/mm holographic diffraction grating (GH25-18V, Thorlabs). The diffracted light is focused by an achromatic doublet lens (AC127-030-B-ML, Thorlabs) into a CMOS detector (ASI294MM, ZWO).

The fluorescence arm of the system is composed of a 485-nm center wavelength LED (20-nm bandwidth) (XPEBBL-L1-0000-00301, Mouser) that illuminates the sample site orthogonally. The fluorescence emission is collected by the same lens used for the Raman emission collection and reflected off a long-pass dichroic mirror (DMLP605, Thorlabs). The fluorescence passes through the long-pass filter (FELH0500, Thorlabs) and is focused into a Raspberry Pi HQ camera using an aspheric condenser lens (ACL12708U-A, Thorlabs). The assembled system on a portable optical breadboard is shown in Fig. 1(b).

### 2.4 Ratiometric SERS/Fluorescence Sample Preparation

A ratiometric SERS/fluorescence sample was developed to validate the multi-modal functionality of the spectroscopic platform. Gold nanoparticles (AuNPs) were synthesized using a seeded growth method adapted from Bastús et al.^49^ Briefly, a 50-ml solution of 2.2-mM sodium citrate (Millipore Sigma) was boiled before the addition of 335  μl of 25-mM ${\mathrm{HAuCl}}_{4}$ (Millipore Sigma). The solution cooled to 90°C forming the gold seed, which was characterized using uultraviolet−visible (UV−Vis) spectrophotometry to determine the size and concentration. The seeded growth protocol facilitates layer-by-layer growth of the AuNPs until the desired diameter is reached. Growth is initiated by pipetting 335  μl of 60-mM sodium citrate to the gold seed solution followed by 335  μl of 25-mM ${\mathrm{HAuCl}}_{4}$ after 2 min. UV−Vis spectrophotometry is performed after each layer growth to monitor the AuNPs solution. The growth layer steps were repeated until the AuNPs reached a size of roughly 34-nm based on the table provided by Haiss et al.^50^ based on the absorbance peak of the AuNPs solution. SERS probes were created by conjugating 4-mercaptobenzoic acid (4-MBA, Millipore Sigma) to the surface of the synthesized AuNPs by shaking them at room temperature for 1 h. Attachment of 4-MBA to the particle surface is facilitated via the thiol moiety. A 4-MBA was chosen as the RRM as it has no fluorescence emission; therefore, fluorescein (Millipore Sigma) was mixed with the SERS probe solution to attain a dual Raman/fluorescence sample. The molar concentration of 4-MBA and fluorescein were inversely varied between 0 and 60  μM to demonstrate that the independent collection paths can distinguish between the two optical modalities without crosstalk.

### 2.5 Multi-modal Assay Preparation

**Fig. 2:**
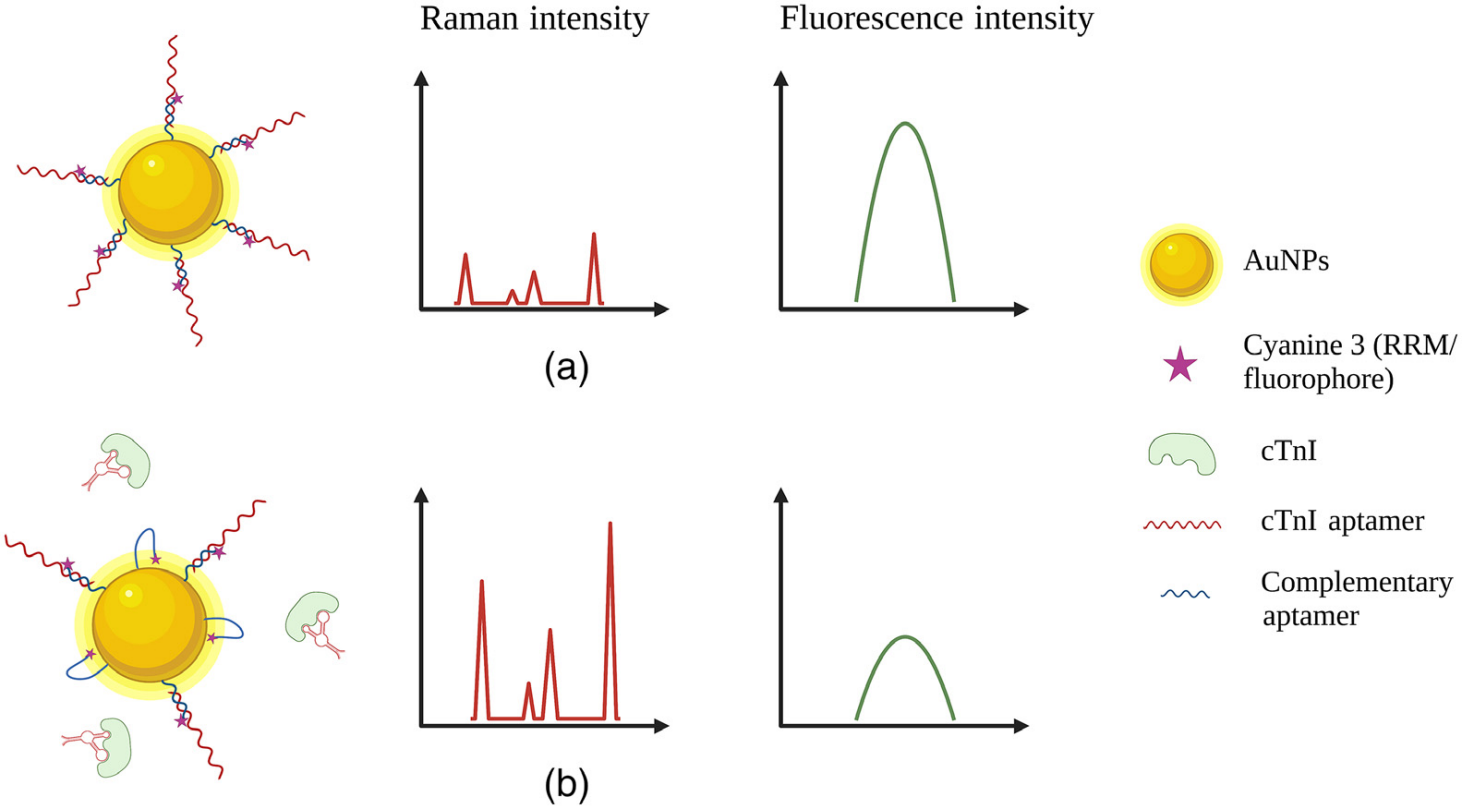
Schematic illustration of the sensing probes and assay response. (a) Denotes the nanoprobes in the absence of cTnI, resulting in a high fluorescence signal and low SERS signal due to the distance between the AuNPs surface and the dual RRM/fluorophore (Cy3). (b) Denotes the probes in the presence of cTnI, resulting in the separation of the hybridized aptamers due to the high binding affinity of the cTnI aptamer to its target. The AuNP-bound complementary aptamer folds to bring the Cy3 molecule closer to the AuNP surface resulting in a decrease in fluorescence signal due to quenching, and an increase in the SERS signal, due to electromagnetic enhancement. (Created with BioRender; see Ref. 53.)

The biosensing ability of the multi-modal spectroscopic platform was demonstrated using a model assay that relies on a conformational change to induce a shift in the spectroscopic signal. The assay was designed to target cardiac troponin I (cTnI), a biomarker commonly used to diagnose acute coronary syndrome. Aptamer sequences used for the assay have been reported in the literature to detect cTnI using electrochemical sensing.^51^^,^^52^ The primary cTnI aptamer sequence is 5′-CGTGCAGTACGCCAACCTTTCTCATGCGCTGCCCCTCTTA-3′. The dual RRM/fluorophore-labeled complementary aptamer sequence is 5′-Cy3-GAAAGGTTGGCGTACT- triethylene glycol spacer-thiol-3′. The aptamer sequences were purchased from Integrated DNA Technologies. The complementary aptamers were incubated at equal volumes with 20-mM tris (2-carboxyethyl) phosphine (TCEP) solution for 1 h to reduce the disulfide bonds into monothiol groups for attachment to AuNPs. The TCEP solution was removed via centrifugation using a 3-kDa nanoseps filter and resuspended in deionization (DI) water. The primary aptamer was filtered using a 3-kDa nanoseps filter and resuspended in DI water. The concentration of the two aptamer solutions was determined via UV–Vis spectrophotometry. The aptamer solutions were heated to 95°C for 5 min before being combined at equal concentrations and cooling to room temperature. The heating allows the aptamers to fold into their tertiary state and hybridize as the solution cools. To remove unhybridized aptamers, the solution was centrifuged using a 10-kDa nanoseps filter, followed by resuspension in DI water. The hybridized aptamer solution and the AuNPs were mixed at a ratio of 6000:1 and allowed to shake for 1 h at room temperature. The solution sat at room temperature overnight to maximize the number of hybridized aptamers to adhere to the AuNPs surface. Salt aging was performed to slowly increase the salt concentration, which helped to decrease the electrostatic repulsion between the aptamers and thus enabled more attachment of the aptamers. About 10  μl of 1 M NaCl was added every hour until the salt concentration reached 50 mM. The solution was left for 12 h and then centrifuged for 15 min at 10 rcf (relative centrifugal force) to remove any unbound aptamers. The probes were then resuspended in a tris buffer (pH 7.4, 50-mM NaCl) and stored in the fridge at 4°C. The developed sensing scheme is shown in Fig. 2.

### 2.6 Device Operation and Data Acquisition

To analyze a sample on the multi-modal platform, a quartz microcuvette (12.5×12.5×45  mm) is inserted into the port on the top of the device. A sample volume of 200  μl is required to ensure irradiation of the sample within the chamber. The data acquisition program triggers the LED to illuminate the microcuvette, and the fluorescence signal is captured with an acquisition time of 10 ms. The LED is then toggled off, the laser diode is triggered to illuminate the microcuvette, and the Raman signal is captured using the CMOS detector with an acquisition time of 15 to 30 s, depending on the sample. The total acquisition time required to obtain the fluorescence and Raman signals sequentially is between 20 and 40 s, including the time it takes the software to toggle between the illumination sources and to collect the optical signals. The LED excitation is executed first to minimize any potential changes in fluorescence signal due to photobleaching that could occur due to the high intensity of the laser diode and the long exposure time for Raman signal collection. A custom macro developed in ImageJ extracts the line across the region of interest for the Raman signal and exports the data to an excel file for further processing. The fluorescence images are processed using a separate macro to extract the intensity plot over the region of interest before exporting to an excel file. The exported Raman data are further processed using an asymmetrical least-squares approach^54^ to remove the background signal.

## 3 Results

### 3.1 Spectrometer Characterization

**Fig. 3:**
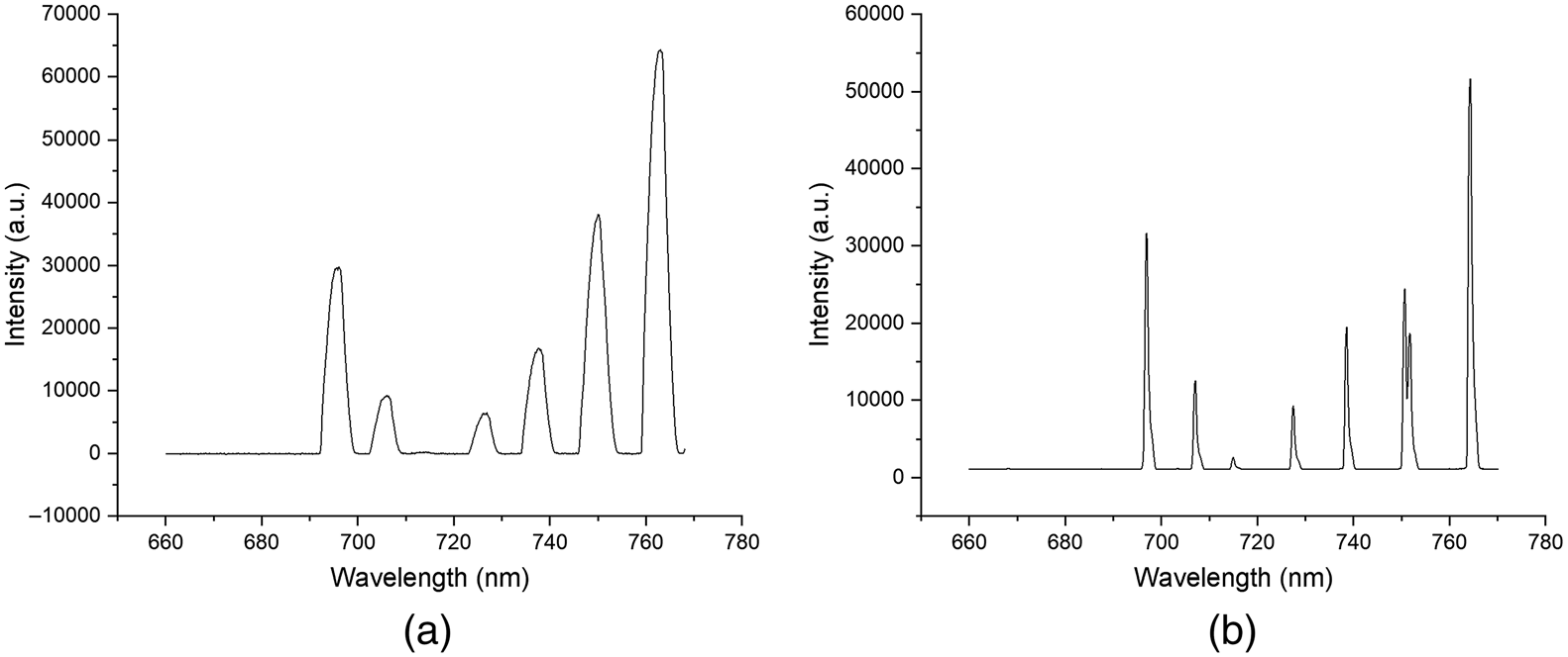
Calibration of the detector area using an Argon emission lamp to correlate pixel location to wavelength. (a) Argon emission spectra were collected from fiber coupling into the commercial USB4000 spectrometer. (b) Argon emission spectra were collected from the developed optical system.

An argon emission lamp was used to calibrate the CMOS detector to register the illuminated pixels with the wavelength of light. A commercial spectrometer (USB4000, Ocean Insight) was used to validate the wavelengths of the emissions peaks and determine the spectral range. Figure 3 shows the argon emission plots between the developed portable optical system and the commercial USB 4000 spectrometer. The spectral resolution of the spectrometer was calculated by measuring the full width at half maximum of the argon emission peaks across the spectral range. The spectral range of the spectrometer was 660 to 770 nm (470 to $2600\;{\mathrm{cm}}^{-1}$) with a spectral resolution of 0.67±0.2  nm. The achieved spectral resolution was slightly lower than the initial design parameters. However, it still provides sufficient resolution of the target Raman peaks for 4-MBA and Cy3 and achieves higher spectral resolution than the commercial system used for calibration (3.69±0.3  nm). Ethanol served as a calibration sample throughout system alignment to validate its ability to efficiently excite and collect Raman scattered light due to its high availability, abundance, and strong Raman emissions.

### 3.2 Multi-modal Validation through Ratiometric SERS/Fluorescence Samples

The multi-modal functionality of the spectroscopic platform was demonstrated using ratiometric SERS/fluorescence solutions. RRM (4-MBA) conjugated AuNPs at the molar concentrations specified in Sec. 2.4 were used to generate a SERS signal, and free-floating fluorescein was used to generate a fluorescence signal. The molar concentrations of each reporter molecule were inversely varied to mimic the behavior of the bioassay. The highest concentration SERS probes were spiked with the lowest fluorophore concentration and vice versa to create an incremental ratiometric gradient across the samples. The portable optical system was benchmarked against two commercial devices: a handheld Raman spectrometer [ID Raman Mini 2.0, Snowy Range Instruments (licensed by Ocean Insight)] for SERS measurements and a Tecan Plate Reader (Infinite 200 Pro) for fluorescence measurements. The ID Raman Mini 2.0 is equipped with a 638-nm laser line, and the Tecan plate reader is equipped with a 485-nm excitation source. Measurements were performed in a standard well plate for the gold standard instruments versus a microcuvette for the developed optical system. To reduce interference from residual dye between different solutions, DI water was used to rinse the microcuvette between samples, and compressed air was used to remove excess water droplets.

**Fig. 4:**
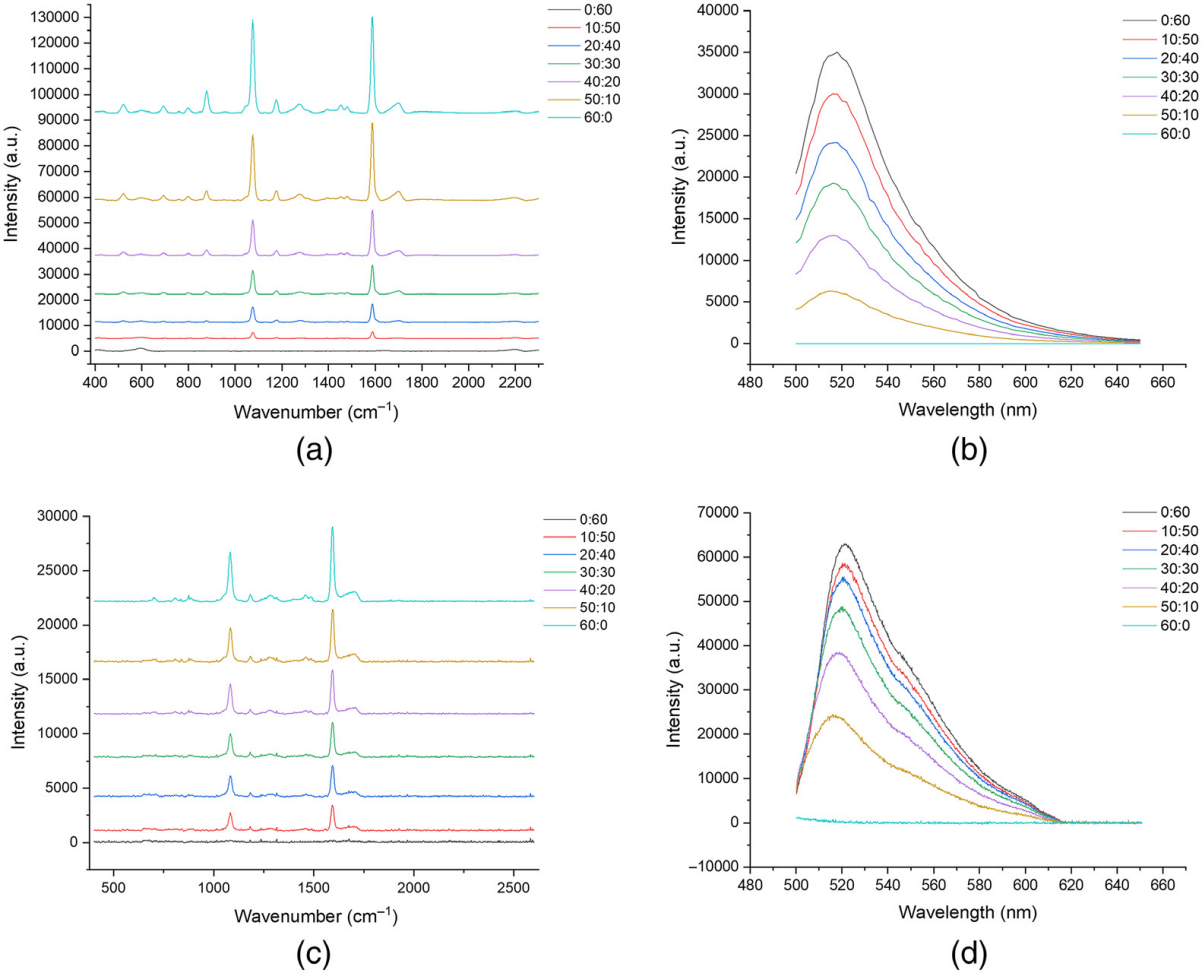
SERS and fluorescence plots from ratiometric RRM/fluorophore solutions compared across the gold standard instruments and the developed optical system. (a) SERS spectra obtained using the ID Raman Mini 2.0 spectrometer (5 s acquisition), and (b) shows fluorescence spectra obtained using the benchtop Tecan plate reader. (c) SERS plot (15 s acquisition time) and (d) shows fluorescence spectra obtained using a multi-modal spectroscopic platform. The fluorescence plot in (d) was obtained by fiber coupling an external spectrometer to the fluorescence collection arm of the developed system to ensure no crosstalk between the collection arms. The concentration ratios denoted in the legends refer to the micromolar concentration of the RRM: the micromolar concentration of the fluorophore in each sample. [Note: the Raman plots of (a) and (c) were purposefully offset for clarity of presentation and they do not actually have different intensity floors.]

**Fig. 5:**
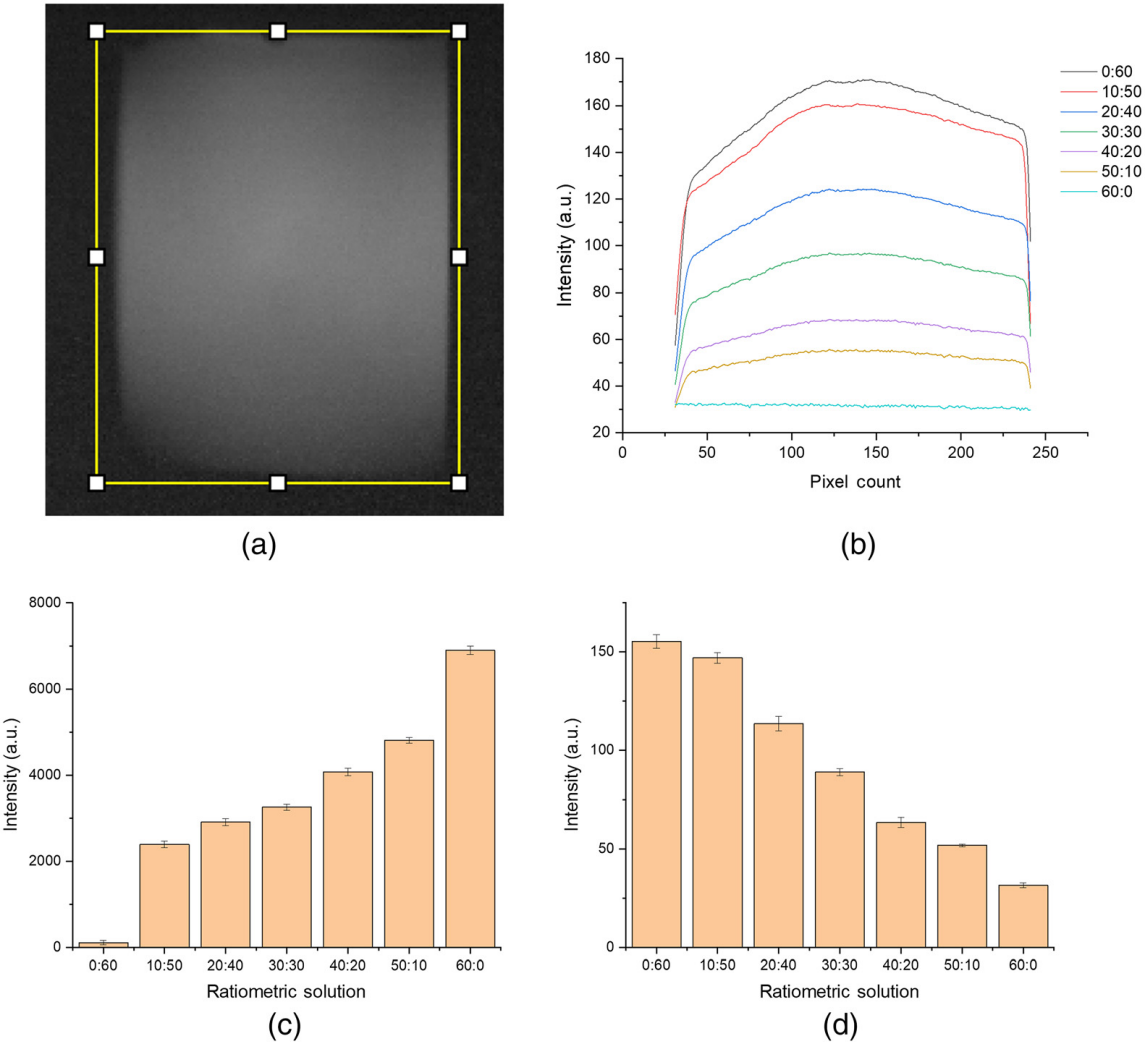
(a) Fluorescence image acquired using the Raspberry PI camera with the yellow box denoting the plotted region of interest. (b) Fluorescence intensity plot for ratiometric SERS/fluorescence solutions collected using the Raspberry PI Camera. The concentration ratios indicated in the legend refer to the micromolar concentration of the RRM: the micromolar concentration of the fluorophore in each sample. (c) SERS peak intensity at $1593\;{\mathrm{cm}}^{-1}$ across the ratiometric SERS/fluorescence solutions shows an increase in the intensity as the concentration of 4-MBA increases. (d) The average fluorescence intensity across the entire field of view for ratiometric SERS/fluorescence solutions shows a decrease in fluorescence intensity as the fluorescein concentration decreases. The concentration ratios on the x-axis refer to the micromolar concentration of the RRM: the micromolar concentration of the fluorophore in each sample.

Due to the orthogonal LED illumination utilized in the optical system for fluorescence excitation, there is the potential for stray light to enter the fluorescence detection arm. To demonstrate that fluorescence emission and not stray LED light were being coupled to the fluorescence arm, a fiber-coupled spectrometer (USB4000) was initially used in place of the Raspberry Pi camera. The fiber-coupled spectrometer was replaced with a Raspberry Pi camera to meet the low-cost design and dynamic range requirements. Figure 4 compares the SERS and fluorescence signals for the commercial instruments versus the developed optical system. The SERS signal shows a linear increase in intensity as the molar concentration increases using the commercial Raman spectrometer and the developed optical system. The same linear trend is observed for the fluorescence signal on both instruments. The plot in Fig. 4(d) shows that the fluorescence emission is being captured with no interference from the LED illumination based on the spectral properties. The fiber-coupled spectrometer was replaced with the Raspberry Pi camera, and the measurements were repeated. Figure 5(a) shows the fluorescence intensity measurements acquired using the Raspberry Pi camera. The fluorescence intensity plot shows the same linear trend demonstrated using the fiber-coupled spectrometer. The plots in Figs. 5(c) and 5(d) show that the Raman and fluorescence signal intensities increase as the molar concentration of each analyte increases within the ratiometric solutions. The images acquired by the Raspberry Pi camera show that the orthogonal illumination cannot evenly illuminate the cuvette volume in the region of interest. As a result, the intensity is lower on the far edge of the cuvette, most distant from the LED, and the illuminated area does not appear as an even rectangle. However, the spectroscopic platform can acquire SERS and fluorescence emissions from a single sample site with no cross-talk between the detection arms.

### 3.3 Multi-modal Validation through Bioassay Testing

The diagnostic utility of the spectroscopic platform was demonstrated using the cTnI bioassay. The aptasensor probes were prepared and added at equal volumes to spiked cTnI samples at various concentrations. The solution was incubated for 30 min at room temperature before taking measurements to allow protein binding. The aptasensor design yields a shift in the spectroscopic signals that correlate to the binding of the biomarker. In the unbound state, the dual RRM/fluorophore on the end of the hybridized aptamers is far from the surface of the AuNPs, resulting in little to no SERS enhancement and negligible fluorescence quenching. Therefore, the initial SERS signal intensity will be low, and the fluorescence signal will be high. In the presence of cTnI, the primary aptamer has a high affinity to the target protein resulting in its dissociation from the AuNP-bound complementary aptamer. The aptamer will then fold, forming a loop hairpin which results in the dual RRM/fluorophore being close to the surface of the AuNPs, resulting in a high SERS enhancement and increased fluorescence quenching. Therefore, the SERS signal intensity will increase, and the fluorescence signal intensity will decrease as cTnI binds to the aptasensor, as shown in Fig. 2. The aptasensor performance was first validated using the same commercial instruments before measuring the multi-modal signal on the developed optical system.

**Fig. 6:**
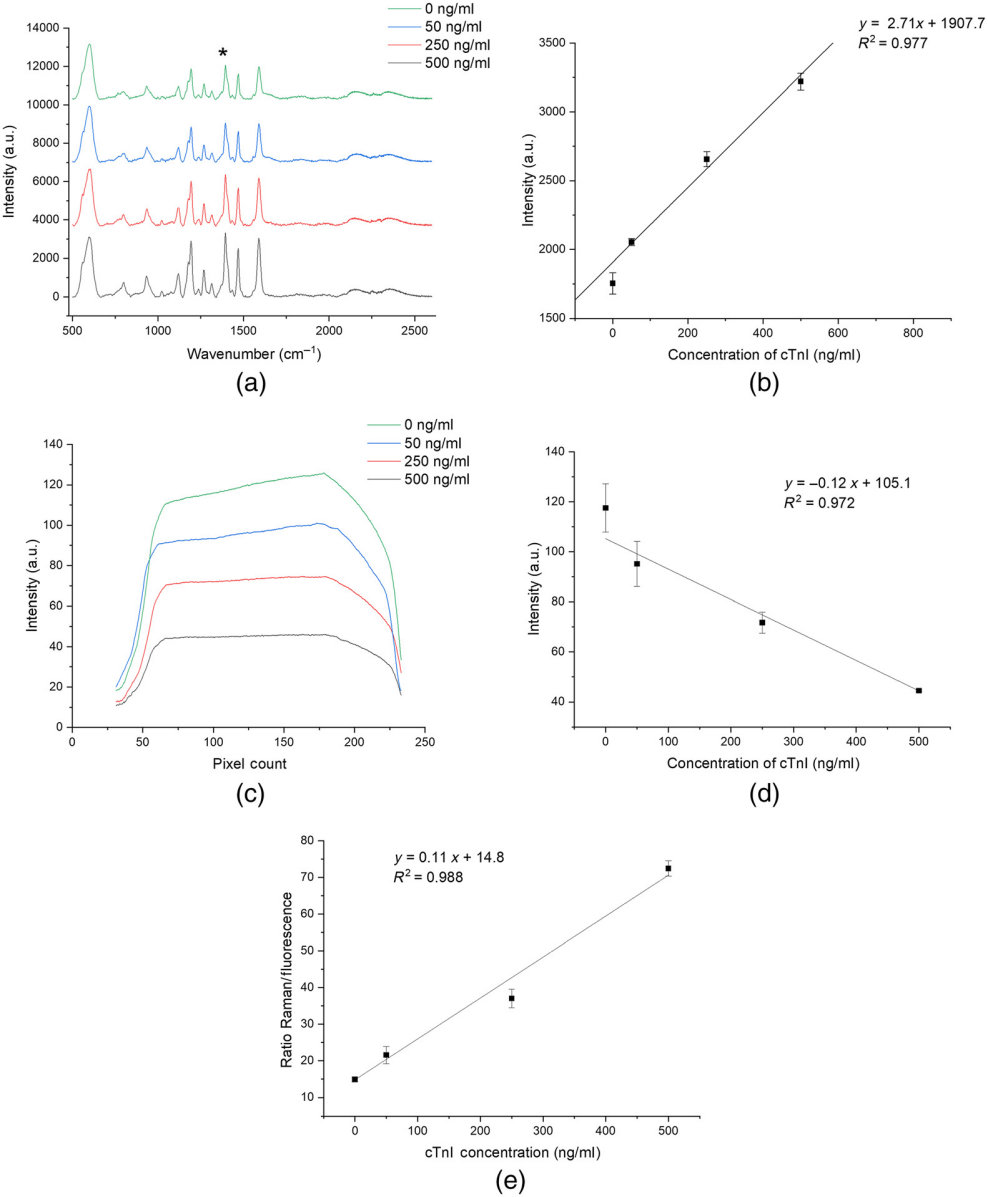
Multi-modal bioassay response to spiked cTnI samples using the multi-modal spectroscopic platform. (a) SERS intensity plot (30 s acquisition) with the * denoting the peak of interest plotted in (b). (b) Linear response of the aptasensor collected from the Raman peak at $1394\;{\mathrm{cm}}^{-1}$. (c) The average fluorescence intensity plot measured across the region of interest corresponds to the illuminated microcuvette area. (d) Linear response of average fluorescence intensity measured as the average across the entire field of view. (e) Linear response of multi-modal approach obtained by dividing the Raman peak intensity at $1394\;{\mathrm{cm}}^{-1}$ by the average fluorescence intensity. Error bars are mean±SD (n=3). [Note: the Raman plots of (a) were purposefully offset for clarity of presentation and they do not actually have different intensity floors.]

The response of the aptasensor (n=3) in the presence of cTnI was measured using the developed multi-modal spectroscopic platform as shown in Fig. 6. The SERS intensity [Fig. 6(a)] increases, and the fluorescence intensity [Fig. 6(c)] decreases as the cTnI concentration increases. The SERS peak intensity at $1394\;{\mathrm{cm}}^{-1}$ and the average fluorescence intensity across the cTnI concentration of 0 to 500  ng/ml show a linear trend in both modalities [SERS: Fig. 6(b) and fluorescence: Fig. 6(d)]. The plotted fluorescence signal is achieved by averaging the pixel intensity across the entire field of view of the acquired microcuvette image. The SERS signal observed for 0  ng/ml could be due to a fraction of the complementary aptamers remaining unhybridized and folded on the surface of the AuNPs. It is important to note that the detected Raman peaks are from the Cy3 molecule, which acts as the RRM/fluorophore and not the aptamers or cTnI. Plotting the Raman peak intensity at $1394\;{\mathrm{cm}}^{-1}$ ratioed against the average fluorescence intensity [Fig. 6(e)] resulted in an improvement in the linear response. We observed that cTnI concentrations between the range from 5 to 10  ng/ml did not result in a lower SERS signal than the 50-ng/ml sample but rather were at a comparable signal intensity and thus indistinguishable. One potential reason for this trend could be that, at lower cTnI concentrations, insufficient aptamers are undergoing the conformational change to elicit a detectable change in the optical signals using the current instrumentation and assay parameters. The fluorescence response [Fig. 6(d)] across the cTnI concentrations showed a higher deviation between samples than the SERS response which could be due, in part, to the fluorescent modality having a high signal intensity in this range and the Raman modality having a low signal intensity in this range, thus the variance in binding led to a bigger signal change. The difference in the intensity scales between the Raman and fluorescence signals also contributes to the higher variance observed in the fluorescence intensity plot and makes a one-to-one comparison challenging. The developed cTnI assay serves as a model bioassay to demonstrate the potential diagnostic utility of the developed optical system. The multi-modal signal acquisition has the potential to improve the detection sensitivity and precision for cTnI or other potential biomarkers.

## 4 Discussion

In this study, we developed a compact form factor multi-modal spectroscopic platform that combines the benefits of fluorescence and Raman including the potential to improve detection sensitivity and accuracy due to the built-in redundancy. The impact of a multi-modal approach on diagnostic sensing is demonstrated using a model aptasensor for cTnI detection. One benefit of the multi-modal approach is highlighted in the improvement of the linearity of the assay response using a ratiometric analysis approach when compared to either optical modality alone. The binding of target biomarkers results in a change in both signals, thus improving the platform’s robustness with the complementary spectroscopic signal acting as a built-in validation metric. This built-in validation metric can also reduce variation between samples, which is often a problem with low concentration biomarkers or single modality approaches. The cTnI aptasensor can be further improved by changing the design of the sensing probes and experimental parameters to achieve signal detection in the clinical range of interest, which is around 0.01 to 0.1  ng/ml. Although the current performance is orders of magnitude away from the clinical range of interest, the study demonstrates a framework that can be employed to detect other biomarkers using the same spectroscopic platform. Previous work in developing dual Raman and fluorescence detection applications highlights its further benefits, including reducing the coefficient of variation between measurements and higher sensitivity and specificity.^38^

The platform’s flexibility enables rapid modifications between different applications in various ways. The front-end optics are currently used to measure from a cuvette. Still, that interface can easily be switched to measure from a paper fluidic or microfluidic platform by (3D) printing a simple mount to attach to the rails on the front of the collection lens. The main limitation is the LED used for fluorescence excitation, which may need to be integrated into the main optical path to illuminate the sample depending on the interface. However, this can be achieved by adding a dichroic mirror to the excitation path. The excitation wavelength and filters can also be swapped with ease due to the use of optical rails to center the optical components with respect to the optical axis. The spectrometer alignment would need to be adjusted to accommodate the change in excitation wavelength, but the use of lenses in the spectrometer body makes the alignment easier to achieve. The desired spectral range can be focused onto the CMOS detector by rotating the fold mirror and grating to meet the requirements for Raman detection. The optics in the fluorescence path can also be easily interchanged to accommodate different excitation wavelengths and emission wavelengths of interest. The Raspberry Pi camera used for fluorescence measurements can also be interchanged with an avalanche photodiode or photomultiplier tube for higher detection sensitivity.

The optical and electrical components of the multi-modal spectroscopic platform are contained in a housing enclosure measuring 12×12×5  in. The data processing program requires an external laptop to output the processed SERS and fluorescence plots. The data processing workflow currently requires two separate programs, but a single program with a user interface can be used to integrate the entire workflow for use at the POC. The optics are mounted using large commercially available mounts that require a secure attachment to the optical breadboard. These mounts can be replaced with 3D-printed optomechanical mounts or injection molded mounts to reduce the overall size and weight of the optical system. The sensitivity needed to perform Raman spectroscopy requires that the components in the spectrometer are secure and unable to shift around during transport or use. Small shifts in the optics can result in misalignment of the system and the inability to collect Raman scattered light efficiently. These modifications improve the robustness of the optical system and bring the spectroscopic platform closer to the final form factor of a POC device.

The spectroscopic platform requires a longer acquisition time (30 s) versus the commercial Raman spectrometer (5 s), which can be reduced by improving light throughput and coupling efficiency into the spectrometer. The fluorescence detection arm utilizes an LED to illuminate the sample cuvette, and the LEDs placement affects the image quality captured by the camera. The cuvette holder has an aperture orthogonal to the microcuvette to allow for orthogonal illumination by the LED. The orthogonal illumination results in decreased signal on the far end of the microcuvette furthest away from the LED source. Adding a collimating lens before the LED can help improve the uniformity of the excitation beam. For diagnostic applications, lower volumes are preferred due to the ability to run multiple tests from a single sample. The commercial instruments utilize a sample volume of around 30  μl versus the 200  μl necessary for the microcuvette, resulting in increased aptasensor probes, protein, reaction time, and overall cost. The dual-modal system can be adapted to collect spectra from microwell plates by redesigning the front optics to address this.

Most of the previous work within the scope of dual Raman and fluorescence detection applications is centered around endoscopic/imaging approaches for spatial localization within diseased tissues, detection of DNA or RNA sequences, and environmental monitoring.^38^^–^^46^^,^^55^^–^^57^ The imaging applications typically utilize fluorescence labeling to determine the general location of target biomarkers within a larger field of view, and the Raman detection is used to discriminate between healthy and diseased regions of interest. The DNA and RNA detection applications focus on developing novel particles, substrates, and sensing schemes to improve detection sensitivity. The environmental monitoring applications utilize high sensitivity probes to detect pollutants in water at low concentrations and have shown promising results that can potentially be translated to biomedical sensing applications. However, these applications still require benchtop confocal microscopes to perform both Raman and fluorescence measurements. The advancements in probe chemistry to enable the generation of multi-modal optical signals has continued to improve over the years. Still, there have been minimal changes in the instrumentation used to carry out the measurements. The developed spectroscopic platform is more suited for sensing and diagnostic applications based on the framework used for the optical design. However, the platform can be modified to meet the needs of imaging applications.

To make an impact in low-resource settings, the developed POC devices need to be portable and provide similar performance metrics to their benchtop counterparts. The spectroscopic platform described here is designed to be mobile with much of the weight contribution coming from the use of an optical breadboard. A significant weight reduction can be achieved by machining a thin sheet of aluminum to match the dimensions of the optical system to reduce the overall weight. Using low-cost optics, off-the-shelf optomechanical mounts, and CMOS detectors within the developed platform allow for sensitive Raman and fluorescence detection at a fraction of the cost of commercial benchtop systems. Within the space of cardiovascular disease, the platform can potentially be used to detect a variety of biomarkers critical for disease detection, including brain natriuretic peptide, creatine kinase-MB, and heart fatty acid binding protein. The developed spectroscopic platform can impact the quality of care within low-resource settings due to its flexibility to be coupled with any number of unique dual Raman and fluorescence probes and assay designs to achieve biomarker detection at the POC.

## 5 Conclusion

A portable, multi-modal spectroscopic platform was developed to collect SERS and fluorescence signals from a single sample site. The optical system utilizes off-the-shelf components and fully integrates with complementary electronics for data acquisition. The multi-modal functionality of the spectroscopic system was validated using ratiometric SERS/fluorescence samples and benchmarked against commercial instruments. The clinical potential of the system was demonstrated using a developed multi-modal aptasensor that relies on conformational change to induce a shift in the optical signals. The work presented shows the potential impact of the developed multimodal spectroscopic platform as a POC diagnostic device to improve the health outcomes of underserved communities.

## Code, Data, and Materials Availability

The data that support the plots within this paper and other findings of this study are available from the corresponding author upon reasonable request.
